# Sex‐ and Age‐Specific Trends in Prescriptions of Beta‐3 Agonists and Anticholinergics in Japan: A Nationwide Prescription Database Study

**DOI:** 10.1111/luts.70092

**Published:** 2026-08-19

**Authors:** Minato Yokoyama, Ayaka Onuki, Kisaki Koshi, Sorami Tsukahara, Haruko Negami, Keiji Shiba, Yuichiro Kato, Akihiro Kojima, Daisuke Kato, Takumasa Amemiya, Tsunehiro Nenohi, Masayasu Urushibara, Motohiro Fujiwara, Touko Asano, Hideto Kano, Kazuhiro Ishizaka

**Affiliations:** ^1^ Department of Urology Teikyo University Hospital, Mizonokuchi Kawasaki Japan; ^2^ Department of Urology Oomori Red Cross Hospital Tokyo Japan; ^3^ Department of Urology Yokohama‐Tsurugamine Hospital Yokohama Japan; ^4^ Department of Urology Nissan Tamagawa Hospital Tokyo Japan

**Keywords:** adrenergic beta‐3 receptor agonists, anticholinergic agents, database, overactive bladder, prescriptions

## Abstract

**Introduction:**

To investigate the nationwide trends in pharmacological treatment for overactive bladder among Japanese adults, specifically focusing on the β3‐agonists to anticholinergics prescribed tablet ratio (BAPR) and how this ratio differs by sex and age.

**Methods:**

National Database Open Data from fiscal year 2015 to 2023 were utilized and the prescription volumes of β3‐agonists (mirabegron and vibegron) and anticholinergics (propiverine, solifenacin, imidafenacin, and fesoterodine) were aggregated. The data were categorized by sex and 10‐year age groups to analyze prescribed tablet trends per 1000 population for each demographic. We calculated the BAPR by dividing the number of prescribed tablets for β3‐agonists by those for anticholinergics and investigated the annual trends for younger and older men and women using an ordinary least squares linear regression model.

**Results:**

During the study period, the total number of prescribed tablets for β3‐agonists, anticholinergics, and overall total showed changes of +285.3%, −24.7%, and +59.3%, respectively. Total and β3‐agonist prescriptions rose annually across all groups, whereas anticholinergic use declined beginning in fiscal year 2018. Additionally, nearly all drugs showed an increase in prescribed tablets per 1000 population as age progressed. Although the BAPR increased significantly in all groups, there were significant differences in the rates of change between groups (*p* < 0.001), with older men exhibiting the highest rate of increase.

**Conclusions:**

A nationwide shift in overactive bladder treatment, where the growth in β3‐agonist prescriptions outweighed the decline in anticholinergics, was observed across all demographic groups in Japan.

## 1 Introduction

Overactive bladder (OAB) is defined as a symptom syndrome characterized by urinary urgency, with or without urgency urinary incontinence, usually with increased daytime frequency and nocturia [1]. Recent research indicates that OAB affects 11.9% of the Japanese adult population, and its prevalence rises with age [2]. While anticholinergics were previously the primary treatment for OAB, there has been a recent shift toward β3‐agonists, a trend that has been demonstrated in several claims‐based studies [3, 4, 5]. Japan was the first country worldwide to launch a β3‐agonist, mirabegron, and with Japan having the largest aging population globally [6], the progress of OAB treatment in Japan will likely serve as a mirror for the future of other nations. To date, there has been only one short communication regarding nationwide trends in OAB pharmacological treatment in Japan [7]. In that study, Suzuki et al. performed an analysis using National Database (NDB) Open Data, which is published annually by the Ministry of Health, Labour and Welfare [8], and allows for various analyses of drug prescription trends. They reported that whereas anticholinergics were prescribed more frequently than β3‐agonists across all age groups in fiscal year (FY) 2015, β3‐agonists surpassed anticholinergics among men aged ≥ 55 years and women aged 70–79 years by FY2020 [7]. However, a study using the US registry database has reported that the ratio of β3‐agonists to anticholinergics as first medication, which had been increasing, has recently declined [4]. Therefore, further investigation is required to clarify the post‐FY2021 trends in Japan, and a more detailed evaluation of these shifts is warranted. In this study, we investigated trends in OAB medications using NDB Open Data, specifically examining how the β3‐agonists to anticholinergics prescribed tablet ratio (BAPR) varied by age and sex.

## 2 Methods

### 2.1 Data Source

NDB Open Data from FY2015 to FY2023 were utilized. From the top 100 items in the urogenital drug category of outpatient clinic data, the number of prescribed tablets to adults aged ≥ 20 years for mirabegron, vibegron, propiverine, solifenacin, imidafenacin, and fesoterodine was aggregated. Although the NDB Open Data has been available since FY2014, it was excluded from the current analysis due to insufficiency, as data from that period were limited to the Top 30 items. Similarly, although data for up to the Top 300 items have been published since FY2022, the NDB Open Data for FY2015 through FY2021 was limited to only the Top 100 items. To ensure consistency across the entire study period, we restricted our analysis to the Top 100 urogenital items. In addition, due to the unavailability of detailed data for imidafenacin in FY2017, data for all prescriptions in FY2017 were omitted from the analysis, because the missing data for imidafenacin have a non‐negligible impact on the interpretation of prescribed tablet trends for OAB and the calculation of BAPR.

### 2.2 Prescribed Tablet Trends for OAB

In addition to overall prescribed tablet trends, we categorized the data by sex and 10‐year age groups to analyze prescribed tablet trends per 1000 population for each demographic group. We calculated the BAPR by dividing the number of prescribed tablets for mirabegron and vibegron by the number of prescribed tablets for propiverine, solifenacin, imidafenacin, and fesoterodine and investigated the annual trends for each sex and age group. Population data were obtained from the national census conducted in October of each year [9].

### 2.3 Statistics

In the present analysis, the study population was divided into eight age groups. Moreover, in the Japanese population, the prevalence of OAB exceeds 10% among individuals aged 60 years and older [2]. Therefore, we used 60 years as the cutoff, which resulted in two groups each below and above 60 years old: younger men, younger women, older men, and older women. To determine the annual rate of change in BAPR and compare longitudinal trends across groups, an ordinary least squares linear regression model with an interaction term was conducted. BAPR was specified as the dependent variable, while FY, modeled as a continuous variable, group (as a categorical variable), and their interaction term (FY × Group) were entered as independent variables. The interaction term was evaluated using analysis of variance to test overall differences in slopes among groups. Upon confirming a statistically significant overall group difference, a post hoc Tukey honestly significant difference test was applied to the estimated slopes. Statistical significance was set at *p* < 0.05. R version 4.2.2 (The R Foundation for Statistical Computing, Vienna, Austria) was used for the analyses.

## 3 Results

**FIGURE 1:**
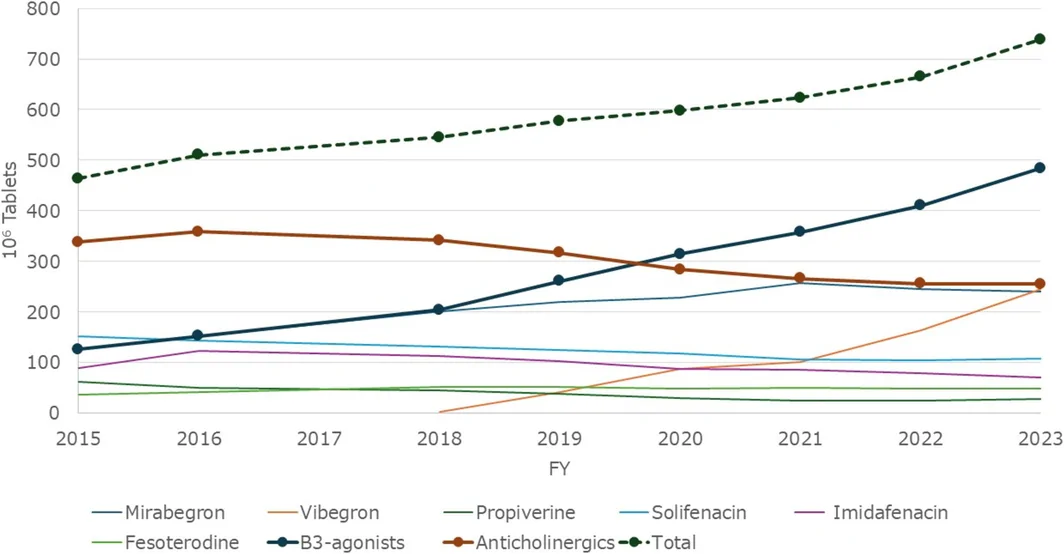
Prescribed tablet trends (millions of tablets per year) of medications for overactive bladder (OAB) in the total population from fiscal year (FY) 2015 to 2023. Data for FY2017 was omitted because of missing data.

In FY2023, the numbers of prescribed tablets were 239.4 million for mirabegron, 244.7 million for vibegron, 28.3 million for propiverine, 108.0 million for solifenacin, 70.7 million for imidafenacin, and 47.5 million for fesoterodine. These figures represented changes of +90.5%, +8202.6%, −53.8%, −29.0%, −20.6%, and +33.4%, respectively, since the initial year of the study (Figure 1). The initial year for vibegron was FY2018, and that for the other drugs was FY2015. The total number of prescribed tablets for β3‐agonists, anticholinergics, and overall total showed changes of +285.3%, −24.7%, and +59.3%, respectively.

**FIGURE 2:**
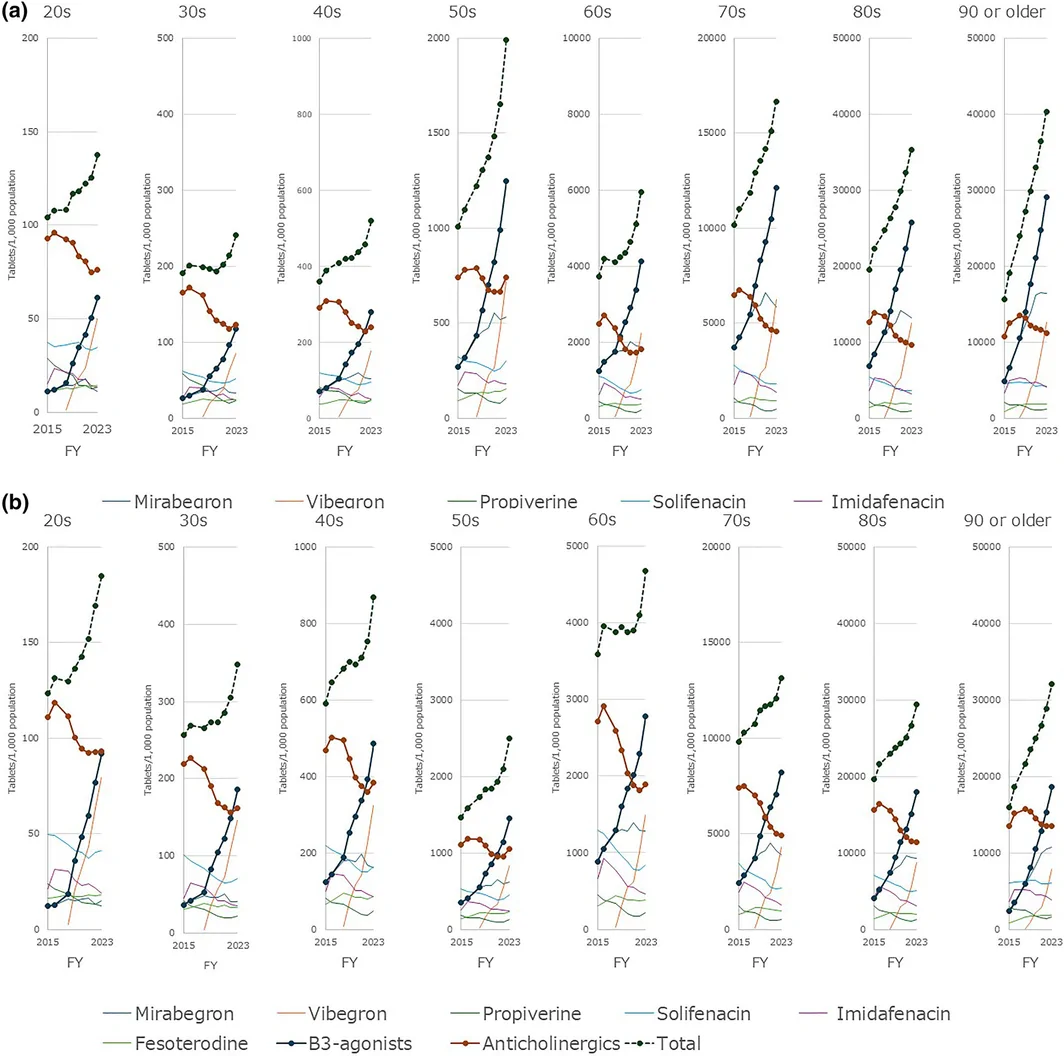
Trends of proportions of prescribed tablets per 1000 population in each age group for (a) men, and (b) women from fiscal year (FY) 2015 to 2023. Data for FY2017 was omitted because of missing data.

Figure 2 presents the number of prescribed tablets per 1000 population, stratified by sex and 10‐year age groups. Total and β3‐agonist prescribed tablets increased across all groups, whereas anticholinergic prescriptions declined. Additionally, nearly all drugs showed an increase in prescribed tablets per 1000 population as age progressed.

**FIGURE 3:**
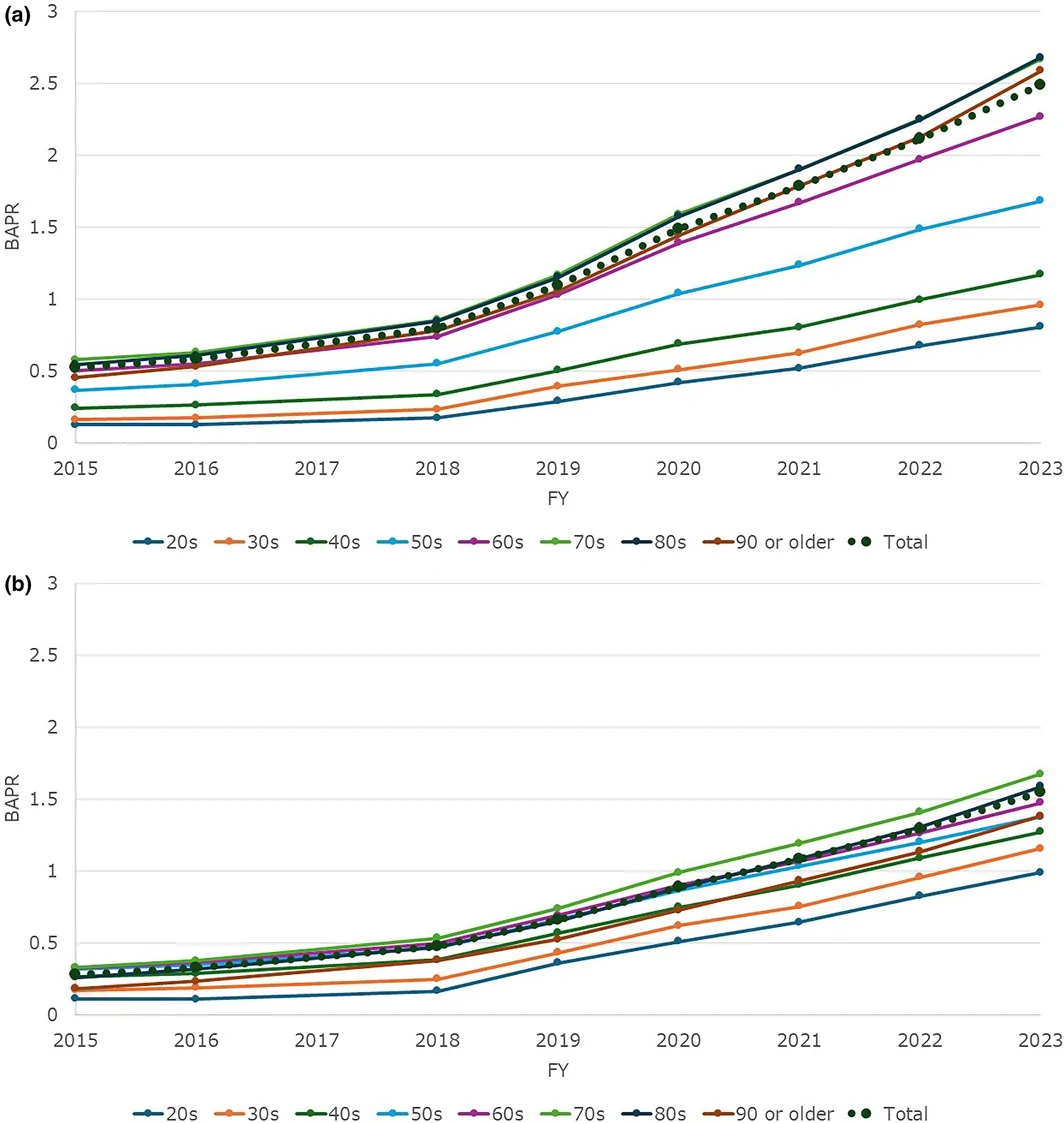
Trend of β3‐agonists to anticholinergics prescribed tablet ratio (BAPR) in each age group for (a) men, and (b) women from fiscal year (FY) 2015–2023. Dotted curves indicate the trend of BAPR in total cohorts. Data for FY2017 was omitted because of missing data.

Whereas the BAPR was less than 1 across all groups in FY2015, it increased annually; by FY2023, it had exceeded 1 in all categories except for men and women in their 20s and men in their 30s (Figure 3). Among women, the ratio increased gradually up to the 70s, before declining slightly in the 80s and 90s or older groups. When comparing age groups, BAPR increased with age up to the 70s, whereas women in their 80s and ≥ 90s showed lower rates than those in their 70s. In males, the BAPR increased with age up to the 80s, with a step‐wise increase observed starting from the 50s.

Linear regression model analysis showed that the annual rate of change in BAPR was +0.160 (95% CI: 0.144–0.176) in the younger men group, +0.130 (95% CI: 0.114–0.146) in the younger women group, +0.252 (95% CI: 0.236–0.268) in the older men group, and +0.158 (95% CI: 0.142–0.174) in the older women group. Although BAPR increased significantly in all groups, there were significant differences in the rates of change between groups (*p* < 0.001). Post hoc pairwise comparisons demonstrated that the slope of the older men group was significantly greater than those of all other groups, and the slope of the younger women group was significantly lower than those of all other groups (all *p* < 0.05, except for the younger men group versus the older women group, *p* = 0.998).

## 4 Discussion

The current study demonstrated trends in OAB pharmacological treatment in Japan. The prescribed tablet volume of OAB medications has been increasing annually, with the volume per 1000 population rising in correlation with advancing age. While the prescribed tablet volume of anticholinergics declined across all age groups from FY2018 onward, it was offset by a larger increase in β3‐agonist prescribed tablets. Consequently, the BAPR has been rising annually, with a particularly steep slope observed among older men. A report from the United States indicated that the ratio of β3‐agonists to anticholinergics as first medication, which had been increasing from 2014 to 2018, shifted to a decline; [4] however, no such trend was observed in the current study in Japan. The difference between Japan and the United States is likely attributable to differences in healthcare and insurance systems.

The prevalence of OAB in Japanese participants aged 40 or older was reported as 12.4% in 2005 [10], which is similar to results of the latest surveillance [2]. Nevertheless, the advancing age of the Japanese population and better awareness of OAB appear to be driving the steady annual rise in prescriptions. The Japanese clinical guidelines for both male and female lower urinary tract symptoms [11, 12] include treatment algorithms tailored for general physicians. Furthermore, some studies from the United States indicated that OAB medications were more frequently prescribed by non‐urologists [13, 14], suggesting that prescriptions from primary care physicians, gynecologists, and other specialists may also be increasing in Japan. However, as the NDB Open Data does not provide information on prescriber specialties, further investigations are required.

Age‐related BAPR trends differed by sex: women showed gradual shifts, whereas men displayed a sharper contrast between younger and older groups. An additional analysis excluding imidafenacin and including the FY2017 data yielded a similar trend (Figure S1). One possible reason is the high prevalence of benign prostate hyperplasia in the older men group. For patients with benign prostate hyperplasia, β3‐agonists would be favored over anticholinergics possibly due to their lower reported risk of inducing urinary retention [15]. Additionally, anticholinergics are associated with a higher incidence of side effects, including dry mouth and constipation. As growing evidence links anticholinergic therapy to increased risks of dementia, falls, and fractures in older patients [16, 17], the tendency to mitigate these risks is likely most evident in the older population. Meanwhile, there remains a certain demand for anticholinergics, for example as prescriptions for patients who do not respond to β3‐agonists and because they are less expensive than β3‐agonists. In particular, they are still prescribed relatively frequently in younger patients.

There are some limitations in this study. First, not all anticholinergics were included in the analysis. This study excluded medications ranked below the Top 100, as well as oral and transdermal formulations of oxybutynin and tolterodine. Although some studies indicated that oxybutynin was the most frequently prescribed anticholinergic for OAB in the United States [3, 4, 5], NDB Open Data showed the relatively small prescription volumes for oral and transdermal oxybutynin and tolterodine in Japan during the study period (data not shown). In fact, the oral oxybutynin and tolterodine dropped out of the NDB Open Data “Top 100” in recent years. Therefore, their exclusion is expected to have only a minimal impact on our overall results. Second, although this study focused solely on the number of prescribed tablets, a certain number of patients were prescribed multiple tablets of a single agent per day; therefore, the actual number of treated patients remains unknown. Additionally, it should be considered that some patients were prescribed both β3‐agonists and anticholinergics simultaneously. Third, this study reflects broad epidemiological prescribing trends based on aggregated database records, rather than patient‐level clinical decision‐making. Because the database lacks individual clinical background information—such as patient comorbidities, symptom severity, or specific medical histories—the underlying medical reasons for the observed prescription shifts remain hypothetical. Therefore, readers should exercise caution when interpreting these shifts, as they represent macro‐level trends rather than direct clinical outcomes.

In conclusion, we observed a nationwide shift in OAB treatment where the growth in β3‐agonist prescribed tablets outweighed the decline in anticholinergics across all demographics. This trend was particularly pronounced in the older male group, showing the greatest rate of change in the BAPR. Despite both drug classes being recommended as first‐line medications in Japanese guidelines, the preferences of physicians and patients have been shifting toward β3‐agonists.

## Author Contributions

**Minato Yokoyama:** conceptualization, data curation, formal analysis, investigation, methodology, writing – original draft preparation. **Ayaka Onuki:** conceptualization, investigation, supervision, writing – review and editing. **Kisaki Koshi:** supervision, writing – review and editing. **Sorami Tsukahara:** supervision, writing – review and editing. **Haruko Negami:** supervision, writing – review and editing. **Keiji Shiba:** supervision, writing – review and editing. **Yuichiro Kato:** supervision, writing – review and editing. **Akihiro Kojima:** supervision, writing – review and editing. **Daisuke Kato:** supervision, writing – review and editing. **Takumasa Amemiya:** supervision, writing – review and editing. **Tsunehiro Nenohi:** supervision, writing – review and editing. **Masayasu Urushibara:** supervision, writing – review and editing. **Motohiro Fujiwara:** supervision, writing – review and editing. **Touko Asano:** supervision, writing – review and editing. **Hideto Kano:** supervision, writing – review and editing. **Kazuhiro Ishizaka:** supervision, writing – review and editing.

## Funding

The authors have nothing to report.

## Disclosure

During the preparation of this work, the authors used Gemini 3 Flash (Google LLC) to improve the English language and readability of the manuscript. After using this tool, the authors reviewed and edited the content as needed and take full responsibility for the final content of the publication.

## Ethics Statement

In accordance with the Ethical Guidelines for Medical and Health Research Involving Human Subjects in Japan, ethical review and informed consent were waived because this study used only publicly available, anonymized aggregate data.

## Conflicts of Interest

The authors declare no conflicts of interest.

## Supporting information


**Figure S1:** Trend of β3‐agonists to anticholinergics prescribed tablet ratio (BAPR) in each age group for (a) men, and (b) women from fiscal year (FY) 2015–2023 excluding imidafenacin and including the FY2017 data. Dotted curves indicate the trend of BAPR in total cohorts.

## Data Availability

The data used in this study are publicly available from the National Database (NDB) Open Data published by the Ministry of Health, Labour and Welfare, Japan (https://www.mhlw.go.jp/stf/seisakunitsuite/bunya/0000177182.html).
